# Feeding practices in public hospitals’ neonatal intensive care units: An exploration into the ways in which COVID-19 affected the best practice in Gauteng

**DOI:** 10.4102/sajcd.v69i2.921

**Published:** 2022-07-22

**Authors:** Kim A. Coutts, Joanne Neille, Nicole Louw

**Affiliations:** 1 Department of Speech Pathology, Faculty of Humanities, University of the Witwatersrand, Johannesburg, South Africa

**Keywords:** COVID-19s, feeding practices, NICU, speech-language pathologists, Gauteng

## Abstract

**Background:**

South Africa’s healthcare system has a multitude of pre-existing challenges prior to the onset of the coronavirus disease 2019 (COVID-19) pandemic, ranging from reduced number of staff, lack of resources and units being at overcapacity both in the adult and paediatric populations. The neonatal intensive care units (NICUs) require a team approach to ensure best practice with vulnerable infants, but little is known about how the onset of the COVID-19 pandemic and the resultant lockdown restrictions impacted the feeding practices within the NICU.

**Objectives:**

This study aimed to explore the impact that COVID-19 had on the feeding practices within the NICU settings in public hospitals in Gauteng.

**Methods:**

A qualitative design was employed with data collected in two NICUs in Gauteng. Data were collected in the form of observations and semi-structured interviews with healthcare workers (HCWs) in the NICU. Data were analysed using inductive thematic analysis.

**Results:**

Although the sample size of participants was limited, social distancing proved to be a challenge resulting in mothers and healthcare workers being given restricted access. This had effects on the ability to provide adequate feeding practices and resulted in anxiety for the mothers and mental health challenges for the HCWs when feeding these at-risk infants. A limitation of this study was the use of only two sites.

**Conclusion:**

COVID-19 amplified the existing challenges in the NICU. A multidisciplinary and family-centred approach to address feeding challenges is required to offset the challenges resulting from the pandemic and subsequent lockdown.

## Background

The coronavirus disease 2019 (COVID-19) pandemic has had a significant impact on healthcare, service provision and the experiences of patients and their families across the globe (Singh & Singh, 2020). Whilst research has emerged pertaining to the impact that the COVID-19 pandemic has had on healthcare, education, financial security and society at large, little is known about the impact that the COVID-19 pandemic has had on feeding practices within the neonatal intensive care unit (NICU). This article strives to explore the impact that the COVID-19 pandemic has had on the implementation of best practice in terms of feeding practices within the NICU from the perspective of the multidisciplinary team (MDT).

The first case of COVID-19 in South Africa (SA) was identified on 05 March 2020, with a national state of disaster being declared on 15 March 2020. On 26 March 2020, SA entered ‘alert level 5’, resulting in many social restrictions, including limiting numbers of people in any form of social gathering, the necessity for the wearing of masks, a restriction on public movement and limited access to public transport. Whilst these measures were necessary to ensure slowing down the spread of the disease and allowing healthcare facilities an opportunity to prepare, they did magnify social inequality (Naidu, 2020) and may have had a negative influence in the feeding practices, bonding and attachment of mothers and neonates born during this period.

Global health policies highlight the importance of the first 1000 days of a child’s life (World Health Organization [WHO], 2002). Whilst this is true for all children, prematurity, birth complications, medical complications, feeding difficulties, challenges to bonding and attachment and admission to the NICU pose significant risks to development and to the establishment of adequate feeding skills, which are necessary to sustain growth and development (Altimeter & Philips, 2016). Where an infant is admitted to the NICU, caregivers may require significant support from the developmental team to bond with their baby and in order to learn to read their communicative signs and signals, as well as to establish good feeding patterns. It is also vital that appropriate feeding practices based on international guidelines are developed and adhered to during this time (Arumigum et al., 2015).

South Africa has an overall neonatal mortality rate of 21 per 1000 live births, with most of these being deaths attributed to pneumonia, diarrhoea and malnutrition (Department of Health, 2013). Rhoda et al. (2018) suggested that some of these deaths may be preventable by ensuring that feeding guidelines, including breastfeeding and safe feeding methods, are explained and demonstrated to caregivers prior to discharge from the hospital. Furthermore, local research suggests that infants return to medical facilities post-discharge with feeding difficulties, which can result in serious complications such as aspiration, poor weight gain and malnutrition (Da Costa et al., 2019). This highlights the need for a coordinated and comprehensive approach to screening, assessment and intervention. Local studies support the need for screening tools to be used in the local NICUs in order to identify at-risk infants (Viviers, Kritzinger, & Vinck, 2016).

Given the complexity of cases and the NICU environment, it is vital to adopt a multidisciplinary approach to assessment and intervention, with caregivers at the centre of the team (Barbosa, 2013; Da Costa et al., 2019). Other team members include but are not limited to doctors, neonatal nurses and other developmental professionals including physiotherapists, occupational therapists, dieticians, social workers, psychologists and speech-language therapists (SLTs). Each team member plays a critical role during the infant’s admission to the NICU; however, there is little research available on the unique role of each team member in establishing optimal feeding practices within the NICUs in the South African public healthcare system (Hardy, Govender, & Naidoo, 2021; Lloyd & De Witt, 2018) and how these roles may have been affected by the COVID-19 pandemic.

Whilst the COVID-19 pandemic necessitated social distancing and at times separation of patients, healthcare providers and family members, the WHO advises against the separation of a mother from her infant, with emphasis placed on the importance of rooming-in and the continuation of the mother providing care to her own newborn infant, which includes maintaining breastfeeding when the mother is able to do so (Calil, Krebs, & De Carvalho, 2020). Whilst research suggests that the chances of transmission of COVID-19 through breastfeeding is minimal (Ahmad et al., 2020; De Miranda et al., 2020), the constant presence of mothers in NICUs is not always possible because of social distancing restrictions. The impact that these restrictions had on infants and their caregivers is yet to be determined.

Given the limited research into the impact that the COVID-19 pandemic has had on infant feeding practices in the NICU, this study aimed to explore the feeding practices in public sector hospital NICUs in the Gauteng province during the COVID-19 pandemic.

## Methods

This article reports on research conducted within two NICUs in tertiary level public hospitals in Gauteng. An exploratory, descriptive design was employed underpinned by structured observations of the NICU and semi-structured interviews with the medical team working in the NICU. In addition, the admissions book was reviewed at both sites to determine the number of admissions relative to the number of nurses working in the NICU, medical diagnoses of infants admitted to the NICU and nature of prescribed feeds.

### Participants

All healthcare workers (HCWs) at both sites were observed during the structured observations and were invited to take part in the interviews. In total, 23 HCWs across both research sites agreed to participate in the semi-structured interviews. Participants are detailed in the Table 1.

**TABLE 1:** Participants included in the study.

Medical professional	Number of interviews conducted (*n*)
Dietician	3
Nurse	6
Nursing assistant	1
Doctor	5
Physiotherapist	1
Speech-language therapist	5
Occupational therapist	2

Inclusion criteria stipulated that participants needed to be working in the NICU and involved in the feeding of infants at the time of data collection. Mothers were excluded from the study because of the nature of the research question and given ethical considerations regarding their vulnerability.

### Data collection

Data were collected over a period of 12 days at site 1 and 5 days at site 2, at which point data saturation was reached. Observations were initially performed and then 14 interviews were conducted at site 1 and eight at site 2, resulting in a total of 23 interviews. Interviews were conducted in a private room or section in close proximity to the NICU, and the length of interviews ranged from 9 min to 38 min.

Two self-developed instruments were used as a guide for the structured observation and the semi-structured interviews. These instruments were created around the themes identified in the literature review and the aims set out for the study. The observational tool was performed following Emerson, Fretz and Shaw’s (2011) guidelines on ethnographic observation. This process involved jotting down notes, building on these to provide elaborate descriptions, thematic analysis of what was observed and reflection on the researchers’ thoughts, feelings, responses and reactions to the observations. The researcher included observations of the following aspects in the tool as described in Table 2.

**TABLE 2:** Themes in the observation tool.

Theme	Subtheme
NICU environment	Rooming-in facilities Noise, lighting, ambience COVID-19 protocols and impact The layout of the unit
Infants	How many infants admitted Reasons for admission
Feeding practices	Who would feed and their role Times they would feed Availability of feeding policies in the unit
Feeding methods	Oral versus non-oral feeds How many babies were on transitioning feeds Breastfeeding in the unit
Feeding types	Formula feeds Breast milk Combination feeds

NICU, neonatal intensive care unit; COVID-19, coronavirus disease.

The researcher constructed a diagram based on these observations that were conducted over a period of a few days. The interview schedule was based on internationally and locally recognised feeding policies (e.g. Department of Health, 2013; UNICEF/WHO, 2016; WHO, 2002) and guided by observations conducted at the two research sites. The nature of questions differed slightly when interviewing the diverse disciplines of professionals because their roles and responsibilities within the NICU all involved different aspects and the interviews maintained a semi-structured approach. Themes for interviews included their description of the NICU and the admitted infants, the unit’s feeding policies and practices, their perceptions on the role of the team and specifically the SLT and lastly, the impact of COVID-19 on the unit and subsequent feeding of infants.

Rigour was ensured through a range of methods including conducting a pilot study prior to beginning data collection, ensuring a sustained presence in the NICU during data collection, triangulation of data collection methods, including a range of professionals in the participant sample and ensuring reliability of thematic analysis with each researcher conducting independent analyses of the observational notes and interview transcripts. No changes needed to be made to the tools based on the pilot study.

Observations took place throughout the day during the period of data collection, with a focus on feeding practices. By focusing on feeding times, it was possible to establish the feeding practices and routines surrounding feeding times and simultaneously identify the team members involved and their dynamics during lockdown restrictions. The diagrams were then completed and the data from the admissions book were recorded.

Individual interviews were carried out face to face and recorded, as this allowed the researcher to listen to all answers and information was provided repeatedly during the process of transcription and analysis. Data obtained from the admission books were entered into a Microsoft Excel spreadsheet, which was stored on an Acer laptop.

### Analysis

Data were analysed using inductive thematic analysis (Braun & Clark, 2006). The transcribed interviews and information on the observational data collection sheets were analysed numerous times to identify the usable data under the codes, and themes were set out. Most of the transcriptions were checked by the second and third authors to ensure reliability.

### Ethical considerations

Ethical clearance was granted through the University of the Witwatersrand Medical Ethics Committee (clearance number: M200220), and from the chief executive officers and unit managers at the two sites. Data collection, analysis and write-up of findings were performed in accordance with the standards stipulated in the Declaration of Helsinki (World Medical Association, 2008). Individual consent was obtained from all participants and assent was obtained from the caregivers.

## Findings and discussion

The findings highlight the ways in which the COVID-19 pandemic amplified existing challenges within the NICU. Findings related specifically to the ways in which the pandemic impacted the NICU environment and the impact of lockdown restrictions on caregivers, staff and logistical arrangements. These themes and subthemes are detailed in Table 3.

**TABLE 3:** Themes and subthemes.

Main theme	Subtheme
NICU environment and feeding practices	Setting Feeding routines Feeding policies Feeding methods
Impact of lockdown restrictions	Social distancing Effect on breastfeeding Family involvement Effect on healthcare workers Carry-over of information

NICU, neonatal intensive care unit.

### The neonatal intensive care unit environment

#### The setting

During our presence in the NICU, the environment was observed to be busy and overstimulating. This was confirmed during the interviews where one nurse (Participant 3) described it as: ‘loud, bright, overcrowded and busy’, and another nurse (Participant 5) said, ‘it should definitely be quiet, more calm and lights down, and less noise, but it’s obviously not’.

The NICUs were often at overcapacity during the data collection period for both sites. Research site 2 was 16% overcapacity at the time of data collection. As observed in the admission books during the observations, which indicated that the ward could take 49 infants, there were over 52 babies admitted. It was also observed during the observations and was confirmed in the interviews by two participants, a nurse (Participant 7) and a SLT (Participant 11), who said ‘because of the influx (overload of infants admitted on a daily), it’s very difficult to follow in the daily routine especially having these very sick babies’ and ‘it is no longer safe, it is overcrowded most of the time. We no longer have a quality set-up that we used to before the overcrowding’ (Participant 7). ‘It’s very busy, uhm, I think everybody works very hard. I think there is not enough staff for all the babies that need to be seen [to]’ (Participant 8, dietician). This impacted the care that was given to the infants.

#### Feeding routines

It was observed that both research sites followed a strict three-hourly feeding routine that started with daily care and monitoring activities. This was confirmed in the interviews by dieticians and nurses. A dietician (Participant 8) mentioned, ‘all babies will be fed three-hourly’. Care activities surrounding feeding times, was explained by a nurse (Participant 1), ‘in that three-hourly routine we change nappies, we feed babies, we do observations, suction if necessary, carry out doctor’s orders and give treatment’. Most other nurses at both sites also mentioned that they do suctioning and check aspirates before every feeding time commences, which is recommended as a feeding tolerance assessment (Yin et al., 2015).

#### Feeding policies

It was observed during the observations that both research sites had a feeding policy on the wall and that the Baby Friendly Initiative (BFI) was followed. However, during the interviews, the HCWs had varying views on feeding policies despite the doctors having a protocol book with the various policies and procedures that were developed by the MDT. These views specifically influenced the transitioning from enteral to oral feeding in terms of when to start and then what to start feeding infants. This was because only 22% of the participants agreed that babies should start breastfeeding prior to discharge.

#### Feeding methods and practices

Regarding the methods of feeding, 40% of infants were fed non-orally during the period of data collection, with the majority of infants at both sites being tube-fed. This was based on the 52 infants observed at the time. These included orogastric and nasogastric tubes. Additional feeding methods included syringe feeding, bottle feeding and intravenous drips. The majority of infants who were fed orally appeared to be fed mixed feeds, consisting of a combination of breast milk and formula feeds, followed by breast milk only, formula milk only and donated breast milk. As a result of the nature of the COVID-19 restrictions, the units were understaffed and at overcapacity during data collection. This resulted in babies being frequently moved around and being discharged, so it was challenging for the researcher to get specific feeding information for each baby based on the observations alone.

Whilst, for most HCWs, the goal was to transition infants who were being fed non-orally onto oral feeds, the restrictions imposed by lockdown regulations, together with reduced capacity and time restrictions, served as the barriers. As a result of social distancing regulations, caregivers were allowed into the NICU on a rotational basis, resulting in feeding becoming the primary responsibility of the nurses as mothers were unavailable. The nurses were observed to have developed feeding methods where they could feed more than one infant at a time because of staff shortages and the units often suffering from overcapacity. Each nurse was responsible for feeding up to 10–15 babies per mealtime as observed during the observations. These feeding strategies involved hanging syringes from drip stands and holding two babies simultaneously as seen during the observations. The long-term impact of these restrictions may have had a negative impact on caregivers being able to exclusively breastfeed, given that the babies were discharged on alternative feeding methods including syringe, cup and bottle feeds.

Babies who were being transitioned onto oral feeds were done so primarily using cup feeding, thus following the BFI as confirmed in the interviews and observations. Some babies were being syringe fed and that was because of resource restrictions. If the mother was present, breastfeeding was encouraged.

### The impact of lockdown restrictions on practices within the neonatal intensive care unit

#### Social distancing in the unit

**FIGURE 1:**
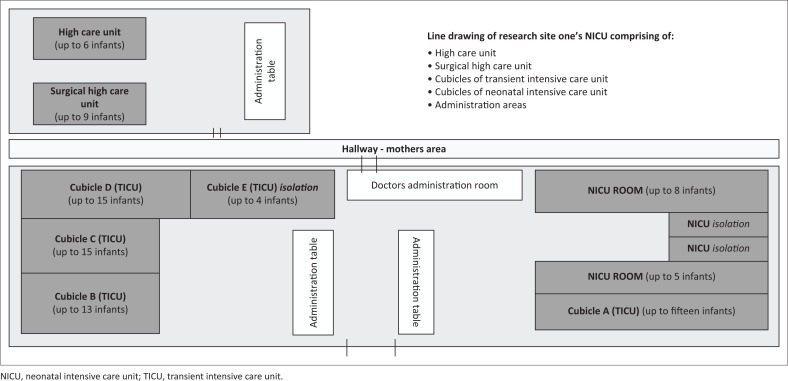
Research site 1 layout.

**FIGURE 2:**
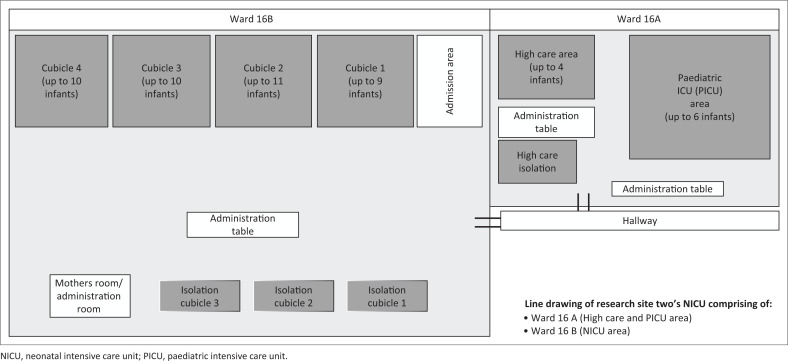
Layout of site 2.

The NICU has always been a setting that follows strict hygiene protocols because of the vulnerability of the infants (Naylor et al., 2020), but as a result of COVID-19 the implementation of distancing between the beds presented a significant challenge. As a result of overcapacity, the recommended 2–3 m distancing between beds could not always be adhered to. Figure 1 and Figure 2 depict the layout of the NICUs in the two different sites.

It is evident from these two diagrams that space is limited within these units, and the implementation of social distancing requirements in a unit that is already at overcapacity presented as a significant challenge. The need for social distancing within the unit also reduced the presence of caregivers and limited staffing capacity. This subsequently impacted the care provided to these infants, especially in terms of how they were being fed. These findings were echoed in a study by Mahoney et al. (2020), who stated that the limitations of parents being involved in the NICU had subsequent impact on the HCWs and the discharge outcomes of these infants. This is problematic because discharged infants would then return to these units for more complications that perhaps could have been avoided. In SA, where the units are already at overcapacity, this needs to be avoided.

#### The effect of COVID-19 on breastfeeding

Research during the pandemic indicated that mothers who tested positive for COVID-19 are still encouraged to breastfeed (Calil et al., 2020). However, as mothers were not allowed into the NICUs where the study was conducted during the harder lockdowns, their breastmilk needed to be expressed so that it could be given to their infants using alternate methods, such as syringe feeding. This was observed during the interviews, where a doctor (Participant 13) said, ‘The mothers will come in to bring in expressed milk. A COVID-19 positive mom, we still allow breast milk to be dropped’. Furthermore, a SLT (Participant 11) observed:

‘Moms were so involved in every part of our therapy, and coming from having moms one day to having practically no moms – only moms that could express and then leave their milk, or moms that eventually when they were lodging were allowed to be here.’

Mothers were not allowed into the NICUs for prolonged periods of time, and many experienced difficulties in accessing the hospital in order to deliver breastmilk because of financial and transport difficulties. It was observed on some occasions that doctors even went to mother’s house to collect the milk, as evidenced where a doctor (Participant 12) said, ‘we had doctors driving from their houses to fetch milk during COVID-19’. By doing so, this placed further pressure on the already overwhelmed medical staff in the overall management of these infants.

#### Family involvement

Pre-COVID-19, both parents and grandparents were allowed into the NICU (McKechnie et al., 2020); however, this changed rapidly at the start of the pandemic. The different sites had varying protocols on who was allowed into the NICU through visiting hours during lockdown. Research site 1 allowed mothers who were breastfeeding their child, but research site 2 allowed all mothers during visiting hours. No other family members, including fathers, were allowed into the NICU at both sites. During an already stressful period, the lack of support for the mothers from their family created further anxiety as described by one of the occupational therapists (Participant 20):

‘The moms are not having the support, I would say. Uhm, because they obviously have to come in but then the dads are not allowed in the hospital grounds at all.’

This was a new finding and requires reflection on how to manage this when moving forward in the pandemic. This lack of family involvement affected not only the medical staff but also had a direct impact on the family as well. These feelings were shared by parents of infants in the NICU in both the Netherlands (Meesters et al., 2021) and in the United States (Vance et al., 2021). Both these studies found that the parents were upset with the decision that the healthcare systems made and that parents needed to be consulted as part of this process. Based on the findings from the study by Meesters et al. (2020) and Vance et al. (2021), a more family-centred approach is required when making important decisions that affect the care of vulnerable infants and their families.

#### Effect of the COVID-19 pandemic on healthcare workers

During lockdown, HCWs were put onto rotations to reduce the number of people present to decrease transmission rates (Mahoney et al., 2020). This was observed in the two research sites. This approach placed pressure on the present HCWs to treat all infants in a unit that was already at overcapacity, as suggested when a dietician (Participant 8) said, ‘in the first wave we had a lot of (COVID-19) positive nurses, and it affected the feeding of the babies and stuff a lot’.

Furthermore, when a staff member tested positive, the whole team was required to quarantine, which affected access to services as revealed when a SLT (Participant 21) said:

‘For us specifically as a department, we had a positive COVID-19 case with one of our audiologists, and so our whole department was shut down for 3 days. As a result there was no one in such a small department to see any of our babies in the NICU.’

This situation resulted in new and often undertrained staff working in highly specialised settings, which affected infant care and HCW performance.

The impact of the pandemic also had a significant impact on staff mental health and performance. This was evident where a doctor (Participant 17) stated, ‘the staff are burnt out and exhausted. And when you are exhausted and burnt out your productivity and functional levels are not as good as, you know, you would’ and where a nurse said:

‘So, we were only three on duty on that day and there were with only two, uhm, what can I say, sessional. Imagine the cubicle with only one nurse and only one assistant in the cubicle.’

‘I was extremely exhausted; my energy level was very low.’

In addition, a SLT (Participant 21) commented:

‘I think the nurses became a lot more stressed and stretched. I think their whole routines were thrown out the window.’

The ways in which the pandemic affected service delivery in the NICU highlights the need for reflection and planning for both the immediate future and long-term in order to ensure that staff mental health is maintained whilst not compromising on service delivery. These findings are supported in other local and international studies (Mahwasane et al., 2020; Pieterse, 2017). A study set in SA specifically highlighted the negative impact of lockdown restrictions had on SLTs and their inability to sufficiently manage patients, especially at an outpatient level in both the private and public sector (Adams, Seedat, Coutts, & Kater, 2021). From this study, the inability to train parents adequately on feeding practices prior to discharge is a significant problem. An inability to train caregivers could result in infants returning to the hospital with other complications (Assad et al., 2016). The information that is required by the family prior to discharge is often missed because HCWs are overworked and are unable to visit the family as often as not.

#### Carry-over of information

Our findings suggest that training on feeding that was performed by various HCWs within the NICU with both parents and nurses was commonplace during pre-COVID-19. The training was performed by multiple members of the MDT. However, with the lockdown restrictions, these services were interrupted as observed in the interviews. A SLT (Participant 11) stated, ‘we also provide training to the nurses. Uhm, it had to stop during COVID-19’, whilst a physiotherapist (Participant 9) added:

‘Obviously we have a role in nurses’ education and obviously with parent education – so parent education came to a halt with COVID-19 and even now we need to be careful of groups.’

The inability to provide the individual treatment plan to the parents and to provide the ongoing guidance and support that parents needed during the NICU stay was a significant finding. A physiotherapist (Participant 9) explained:

‘I think we have missed having the mothers, because if you have a more sort of a long-term baby, we would rely on the mom to carry over what we have been doing. It not only affects the treatment here but the fact that I don’t have a mom to give input to means that when she goes home or when this baby goes home, so she will then just come and get her baby in order to go home. And so she hasn’t had those two or three bits of input. Part of early intervention is actually connecting with the moms. So when COVID-19 hit and the moms were no longer coming in, I would say yes, our practice changed a lot.’

This finding is important and can have lasting effects for when the infant is discharged. The impact of HCWs not being able to have consistent contact with the family resulted in more post-discharge complications as seen by Meesters et al. (2020). A long-term study on the impact of COVID-19 on infants during this time in SA is perhaps warranted.

**FIGURE 3:**
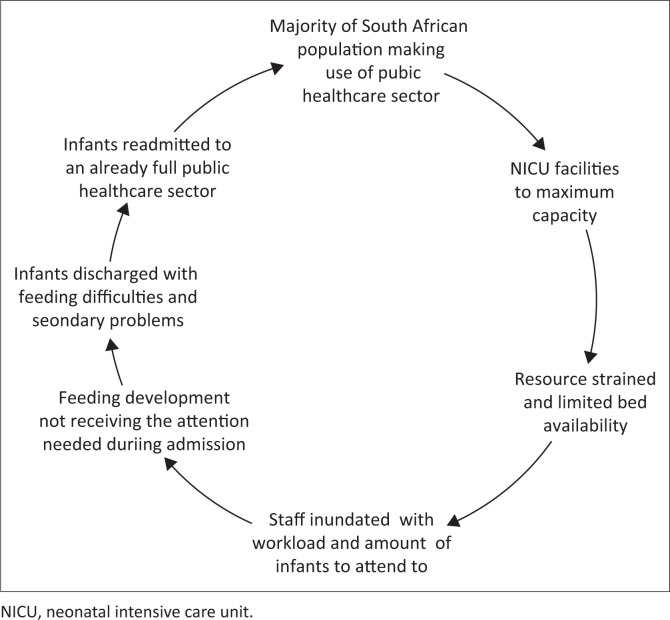
Feeding challenges within the neonatal intensive care unit prior to COVID-19.

These findings suggest that existing challenges in the NICU prior to the COVID-19 pandemic were mostly related to the units being at overcapacity, understaffing of HCWs in the unit and limited resources as reflected in Figure 3.

These ongoing challenges as depicted in Figure 3 were amplified during the pandemic. There were also new challenges found, such as the impact that new policies had on HCWs and families. This study further supports the notion that feeding is a complex and multifaceted process that, despite lockdown restrictions, requires MDT input within a family-centred approach to ensure ongoing support and reduced complications (Green et al., 2021; Pike et al., 2016; Younesian, Yadegari, & Soleimani, 2015). In the case where restrictions limit the number of HCWs present, the need for relying on other HCWs becomes important to assist in certain areas in terms of method of feeding. This suggests the need for considering a revision on practice patterns, such as a transdisciplinary approach, is required to offset these challenges in the NICU. This type of approach has been suggested in adult dysphagia cases in a variety of settings to offset certain resource challenges (Coutts, 2021).

The types of feeds that infants are receiving, whether it be formula or mixed feeds, was because of the decreased presence of mothers and the transport challenges that they experienced in needing to get their expressed milk to the hospital. This could lead to intolerances if mothers wanted to change types of feeds at home (Assad et al., 2016). This is a potential challenge that can lead to infants returning post discharge for more care. In the SA setting, this needs to be avoided.

Overall, family members should be involved more consistently, despite the level of lockdown restrictions, and should be supported throughout the process. This can assist with reducing the load on HCWs, reduce parental anxiety and possibly reduce the number of infants that might return with complications.

## Conclusion

This study proved that COVID-19 had a significant negative influence on the already burdensome public healthcare sector in SA. It confirmed that NICUs are at overcapacity and the shortage of HCWs influenced staff’s mental health and the subsequent care provided in terms of feeding to both the infants and their parents. Prior to COVID-19, support to mothers was already a challenge due to limited staff and wards being at their maximum capacity, and the COVID-19 merely exacerbated these challenges, which created potential complications when infants were being discharged.

These environments need an increase in HCWs to provide the services needed by the number of infants in this setting, when it comes to adequate feeding practices of these at-risk infants. With more dedicated HCWs within the NICU, the feeding development of infants can be supported optimally. Infants can also be guided safely to discharge successfully, even during lockdown restrictions. Another possible solution and research recommendation would be to implement a more transdisciplinary approach to the management of feeding with the NICUs during these circumstances, and how to better utilise mothers within the NICU environment to assist with feeding. Further investigation on the longer effects that COVID-19 has had on this setting, the HCWs, infants and families is also required.

## References

[CIT0001] Adams, S., Seedat, J., Coutts, K., & Kater, K. (2021). ‘We are in this together’ voices of speech-language pathologists working in South African healthcare contexts during level 4 and level 5 lockdown of COVID-19. *South African Journal of Communication Disorders*, 67(1), a792. 10.4102/sajcd.v68i1.792 PMC8007987 33764150

[CIT0002] Ahmad, K.A., Darcy-mahoney, A., Kelleher, A.S., Ellsbury, D.L., Tolia, V.N., & Clark, R.H. (2020). Longitudinal Survey of COVID-19 Burden and Related Policies in U.S. Neonatal Intensive Care Units. *American Journal of Pe rinatology*, 38. 10.1055/s-0040-1718944 PMC7869047 33075846

[CIT0003] Altimier, L., & Phillips, R. (2016). The Neonatal Integrative Developmental Care Model: Advanced Clinical Applications of the Seven Core Measures for Neuroprotective Family-centered Developmental Care. *Newborn and Infant Nursing Reviews*, 16(4), 230–244. 10.1053/J.NAINR.2016.09.030

[CIT0004] Arumugam, V., Arunan, S.K., Balasubramanian, G.P., & Parachuri, S. (2016). A prospective study on medication and total parenteral nutrition practices at a Neonatal Intensive Care Unit. *Archives of Pharmacy Practice*, 7(4), 142–148.

[CIT0005] Barbosa, V.M. (2013). Teamwork in the Neonatal Intensive Care Unit. *Physical & Occupational Therapy in Pediatrics*, 33(1), 5–26. 23311520 10.3109/01942638.2012.729556

[CIT0006] Braun, V., & Clark, V. (2006). Using thematic analysis in psychology. *Qualitative Research in Psychology*, 3(2), 77–101.

[CIT0007] Calil, V.M.L.T., Krebs, V.L.J., & De Carvalho, W.B. (2020). Guidance on breastfeeding during the Covid-19 pandemic. *Revista da Associacao Medica Brasileira*, 66(4), 541–546. 10.1590/1806-9282.66.4.541 32578793

[CIT0008] Coutts, K.A. (2021). Dysphagia in cervical spinal cord injury: How international literature trends can guide South African practice patterns – A scoping review. *South African Journal of Physiotherapy*, 77(1), 7. 10.4102/sajp.v77i1.1542 PMC8182465 34192210

[CIT0009] Da Costa, M.A., Kruger, E., Kritzinger, A., & Graham, M.A. (2019). Prevalence and associated prenatal and perinatal risk factors for oropharyngeal dysphagia in high-risk neonates in a South African hospital. *South African Journal of Communication Disorders*, 66(1). 10.4102/sajcd.v66i1.637 PMC6890542 31793313

[CIT0010] Department of Health. (2013). *Infant and young child feeding policy*. Retrieved from https://www.health-e.org.za/wp-content/uploads/2013/09/IYCF_Policy_2013.pdf

[CIT0011] De Miranda, V.S.G., Rech, R.S., Maahs, M.A.P., Berbert, M.C.B., & de Almeida, S.T. (2020). Speech therapy, breastfeeding and COVID-19: information to speech therapist. *Codas*, 32(3). 10.1590/2317-1782/20192020124 32428084

[CIT0012] Emerson, R., Fretz, R., & Shaw, L. (2011). *Writing ethnographic field notes* (2nd ed.). Chicago, IL: University of Chicago Press.

[CIT0013] Green, J., Staff, L., Bromley, P., Jones, L., & Petty, J. (2021). The implications of face masks for babies and families during the COVID-19 pandemic: A discussion paper. *Journal of Neonatal Nursing*, 27, 21–25. 33162776 10.1016/j.jnn.2020.10.005 PMC7598570

[CIT0014] Hardy, M., Govender, P., & Naidoo, D. (2021). Novice occupational therapist’s experience of working in neonatal intensive care units in KwaZulu-Natal. *South African Journal of Occupational Therapy*, 51(1), 27–35. 10.17159/2310-3833/2021/vol51n1a5

[CIT0015] Lloyd, L.G., & Witt, T.W. de. (2018). Neonatal mortality in South Africa: How are we doing and can we do better? *South African Medical Journal*, 103(8), 518–519. 10.7196/SAMJ.7200 23885729

[CIT0016] Mahoney, A.D., White, R.D., Velasquez, A., Barrett, T.S., Clark, R.H., & Ahmad, K.A. (2020). Impact of restrictions on parental presence in neonatal intensive care units related to coronavirus disease 2019. *Journal of Perinatology*, 40, 36–46. 32859963 10.1038/s41372-020-0753-7 PMC7453850

[CIT0017] Mahwasane, T., Maputle, M.S., Simane-netshisaulu, K.G., & Malwela, T. (2020). Provision of Care to Preterm Infants at Resource Limited Health Facilities of Mopani District, South Africa. *Annals of Global Health*, 86(1), 1–8. 32064228 10.5334/aogh.2555 PMC7006596

[CIT0018] McKechnie, L., MacSween, K., Fraser, C., Clinton, T., Clements, D., & Patel, N. (2020). The Impact of the Current SARS-CoV-2 Pandemic on Neonatal Care. *Infant*, 16(8), 134–137.

[CIT0019] Meesters, N., Van Dijk, M., Carvalho, F.S. de., Haverman, L., Reiss, I.K.M., Simons, S.H.P., & Van den Bosch, G.E. (2021). COVID-19 lockdown impacts the wellbeing of parents with infants on a Dutch neonatal intensive care unit. *Journal of Pediatric Nursing*, 62, 106–112. 10.1016/j.pedn.2021.09.024 34642075 PMC8482115

[CIT0020] Naidu, T. (2020). The COVID-19 pandemic in South Africa. *Psychological Trauma: Theory, Research, Practice and Policy*, 5(12), 559–561. 10.1037/tra0000743 32525389

[CIT0021] Pieterse, C. (2017, May 29). Staff speaks out at Edendale hospital. The Witness. https://www.news24.com/SouthAfrica/News/staff-speak-out-at-edendale-hospital-20170528

[CIT0022] Pike, C., Pike, M., Kritzinger, A., Krüger, E., & Viviers, M. (2016). Risk profiles of infants ≥ 32 weeks ’ gestational age with oropharyngeal and oesophageal dysphagia in neonatal care. *South African Journal of Child Health*, 10(2), 130–133.

[CIT0023] Rhoda, N., Velaphi, S., Gebhardt, G., Kauchali, S., & Barron, P. (2018). Reducing neonatal deaths in South Africa: Progress and challenges. *South African Medical Journal*, 108(3a), s9–s16. 10.7196/SAMJ.2017.v108i3b.12804

[CIT0024] UNICEF/WHO. (2016). *Updates on HIV and infant feeding*. Retrieved from https://apps.who.int/iris/bitstream/handle/10665/246260/9789241549707-eng.pdf?sequence=1

[CIT0025] Vance, A.J., Malin, K.J., Miller, J., Shuman, C.J., Moore, T.A., & Benjamin, A. (2021). Parents’ pandemic NICU experience in the United States: A qualitative study. *BMC Pediatrics*, 21(1), 558. 10.1186/s12887-021-03028-w 34886824 PMC8655088

[CIT0026] Visser, M., & Nel, M. (2016). Breastfeeding among mothers in the public health sector: The role of the occupational therapist. *South African Journal of Occupational Therapy*, 46(2), 65–72. 10.17159/2310-3833/2016/v46n2a11

[CIT0027] Viviers, M., Kritzinger, A., & Vinck, B. (2016). Development of a clinical feeding assessment scale for very young infants in South Africa. *South African Journal of Communication Disorders*, 63(1), 11. 10.4102/sajcd.v63i1.148 PMC5843192 27796101

[CIT0028] World Health Organization (WHO). (2002). *Infant and young child nutrition: Global strategy on infant and young child feeding*. Retrieved from http://apps.who.int/gb/archive/pdf_files/WHA55/ea5515.pdf?ua=1

[CIT0029] World Health Organization (WHO). (2019) *Newborns: Reducing mortality*. Retrieved from https://www.who.int/news-room/fact-sheets/detail/newborns-reducing-mortality

[CIT0030] World Medical Association. (2008). *World Medical Association declaration of Helsinki: Ethical principles for medical research involving human subjects*. Ferney-Voltaire: World Medical Association.

[CIT0031] Yin, L., Qian, L., Zhu, H., Chen, Y., Li, H., Han, J., & Qiao, L. (2015). Application effect of extensively hydrolyzed milk protein formula and follow-up in preterm children with a gestational age of less than 34 weeks: Study protocol for a randomized controlled trial. *Trials*, 16, 498. 10.1186/s13063-015-1030-5 26537897 PMC4632355

[CIT0032] Younesian, S., Yadegari, F., & Soleimani, F. (2015). Impact of oral sensory motor stimulation on feeding performance, length of hospital stay, and weight gain of preterm infants in NICU. *Iranian Red Crescent Medical Journal*, 17(7), e13515. 26421163 10.5812/ircmj.17(5)2015.13515 PMC4583832

